# Implementation contexts and the impact of policing on access to supervised consumption services in Toronto, Canada: a qualitative comparative analysis

**DOI:** 10.1186/s12954-019-0302-x

**Published:** 2019-05-02

**Authors:** Geoff Bardwell, Carol Strike, Jason Altenberg, Lorraine Barnaby, Thomas Kerr

**Affiliations:** 1 British Columbia Centre on Substance Use, 400-1045 Howe Street, Vancouver, BC V6Z 2A9 Canada; 2 Department of Medicine, University of British Columbia, 400-1045 Howe Street, Vancouver, BC V6Z 2A9 Canada; 3 Social and Behavioural Health Sciences, Dalla Lana School of Public Health, University of Toronto, 155 College Street, Toronto, ON M5T 3 M7 Canada; 4 South Riverdale Community Health Centre, 955 Queen Street East, Toronto, ON M4M 3P3 Canada; 5 Parkdale Queen West Community Health Centre, 168 Bathurst Street, Toronto, ON M5V 2R4 Canada

**Keywords:** Supervised consumption services, Supervised injection sites, Police, Surveillance, Implementation context

## Abstract

**Background:**

Supervised consumption services (SCS) are being implemented across Canada in response to a variety of drug-related harms. We explored the implementation context of newly established SCS in Toronto and the role of policing in shaping program access by people who inject drugs (PWID).

**Methods:**

We conducted one-to-one qualitative semi-structured interviews with 24 PWID. Participants were purposively recruited. Ethnographic observations were conducted at each of the study sites as well as in their respective neighbourhoods. Relevant policy documents were also reviewed.

**Results:**

Policing was overwhelmingly discussed by participants from both SCS sites. However, participant responses varied depending on the site in question. Subthemes from participant responses on policing at site #1 described neighbourhood police presence and fears of police harassment and drug arrests before, during, or after accessing SCS. Conversely, subthemes from participant responses on policing at site #2 described immunity and protection from police while using the SCS, as well as a lack of police presence or fears of police harassment and arrests. These differences in implementation contexts were largely shaped by differences in local neighbourhoods and drug scenes. Police policies highlighted federal laws protecting PWID within SCS, but also the exercise of discretion when applying the rule of law outside of these settings.

**Conclusions:**

Participants’ perspectives on, and experiences with, policing as they relate to accessing SCS were shaped by the implementation contexts of each SCS site and how neighbourhoods, drug scenes, and differences in policing practices affected service use. Our findings also demonstrate the disconnect between the goals of policing and those of SCS. Until larger structural barriers are addressed (e.g. criminalization), future SCS programming should consider the impact of policing on the SCS implementation context to improve client experience with, and access to, SCS.

## Introduction

Supervised consumption services (SCS) have been implemented in Australia, Europe, and North America in response to a variety of drug-related harms such as overdose and infectious disease transmission [1–5]. Sometimes referred to as drug consumption rooms or safe injection sites, international studies have demonstrated the effectiveness of these public health interventions in reducing harms for people who inject drugs (PWID). For example, research has shown reductions in overdose mortality and overdose-related ambulance calls during the operating hours of SCS [6–8]. SCS have also led to a decrease in syringe reuse and sharing among PWID [9, 10]. Furthermore, SCS have connected individuals to treatment, such as detoxification services and opioid agonist treatments [11, 12]. Aside from the benefits of SCS for PWID, there are broader positive outcomes of these services for the general public, including decreases in public injecting practices and publicly discarded drug equipment [9, 13–15], and potential negative impacts such as increases in drug-related activities in the neighbourhoods surrounding SCS have not been observed [16–18].

While numerous benefits of SCS have been observed internationally, harm reduction interventions have faced opposition from a variety of stakeholders, including some governments [19, 20], community stakeholders [21, 22], and police [23]. Given the criminalization of drugs and PWID in multiple jurisdictions internationally, police have played a prominent role in shaping access to a variety of harm reduction programs [24]. For example, PWID in multiple settings have experienced the confiscation of drug paraphernalia (e.g. needles, syringes, cookers) by police [25–27], sometimes resulting in arrest, which has been associated with syringe sharing and public injecting [28]. Public surveillance and policing has led PWID to rush their drug use [29, 30] and has produced persistent fears of arrests, extortion, and violence [31]. Research has also found associations between intensified policing and an increased risk of infectious disease transmission due to syringe sharing [32]. Furthermore, studies have shown how police encounters act as barriers to accessing health services, including methadone maintenance treatment [33], adherence to highly active antiretroviral therapy [34], and needle and syringe programs [35].

Given that criminalization and policing have had a negative effect on PWID, some studies have suggested potential benefits of building more productive relationships between police and harm reduction interventions [36–38]. In the Canadian context, stakeholder perspectives are important to consider for the implementation of SCS, as federal laws that govern the approval and implementation of these services require community consultations [19, 39]. Included in these community consultations are police departments, who have often been in opposition to SCS [23]. Police in the city of Toronto, however, have more recently changed their position on these services, with the police chief publicly voicing his support for SCS [40]. Police support for SCS has been integral for the approval and implementation of SCS in Toronto [40], as well as other harm reduction services such as needle and syringe programs across the province of Ontario [41]. However, while a recent international study examined the development of cooperative relationships between police and SCS management, including in Toronto [38], given the realities of drug criminalization and the negative experiences that people who inject drugs (PWID) have had with police, it is not known how the practice of policing in different Toronto neighbourhood contexts may impact SCS client uptake.

While SCS have been shown to improve various individual and community health outcomes, these services do not exist in isolation and also need to be considered in relation to the contexts (e.g. geographical, legal) in which they are being implemented. For example, research has shown that a lack of consideration for the contexts of HIV/AIDS interventions increases their likelihood to fail or underperform [42]. Studies in implementation science, therefore, have increasingly considered the role that context plays in affecting the implementation of health interventions [43, 44]. Considering contextual domains alongside public health interventions such as SCS thus provides a more robust understanding of the interventions’ implementation challenges and outcomes, including those specific to each intervention location.

Two newly established SCS have been implemented in community health centres (CHCs) in two distinct Toronto neighbourhoods. Site #1 is located in a traditionally working class and immigrant neighbourhood. The neighbourhood is a mixed-use area that includes residential, commercial, and numerous health and social services (e.g. drop-in centres, shelters, hospitals). Over the last decade, the area has experienced gentrification via condominium and student housing development, which has resulted in a significant increase in rental costs, and consequently the displacement of low-income communities. Despite gentrification, there is still a concentration of people who are homeless and experience ongoing housing instability. Open drug use continues to occur both outdoors in the surrounding area and indoors (e.g. public washrooms, business establishments, social service agencies). As a result, there is a significant police presence in the surrounding area. The CHC along with health and social services works collaboratively with the local Toronto Police division to address community concerns regarding the neighbourhood and SCS. Additionally, SCS staff provide education to frontline police officers regarding their services. Site #2 is historically a working class and industrial neighbourhood. Similar to site #1, this area has experienced gentrification. Despite this reality, the neighbourhood continues to have a concentration of social housing and shelters that sustain service needs. A decade ago, public drug use (e.g. parks, laneways) was commonplace; however, in the last 5 years, the nature of public drug use has shifted to more indoor spaces (e.g. libraries, local service agencies, social housing units). The CHC has offered harm reduction and health services since 1998 and has worked collaborative with the local Toronto Police division since its inception. There is a general consensus that local police are supportive of their harm reduction services and recognize the limited effectiveness of criminalization of PWID. In this study, we compare and examine the implementation contexts of these two SCS and the role of policing in shaping program access by PWID.

## Methods

Rapid ethnographic data collection occurred over a 1-week period in late July 2018. Qualitative methods, including individual semi-structured interviews and ethnographic observation, were employed at two SCS located in distinct Toronto neighbourhoods. The interviews sought to capture participants’ drug use experiences as well as barriers and benefits to accessing SCS. Ethnographic observation was used at each of the SCS sites and within their respective neighbourhoods. Ethical approval was obtained through the University of British Columbia/Providence Health Care Research Ethics Board.

For the individual interviews, we purposively sampled 24 PWID from the SCS sites (12 from each site). Recruitment of participants ended once saturation in data collection was complete at each site. Participants were required to have used SCS at one of the study sites at least once. The lead author recruited potential participants via the SCS as well as from the surrounding neighbourhoods (e.g. drop-in spaces). The lead author kept a record of demographic information to ensure a diverse representation of participants in terms of gender and ethnicity. Participants provided written informed consent and received $30 CAD cash honoraria. An interview guide was used to facilitate the interviews and focused on drug use and individual experiences using SCS as well as contextual factors that may impact the use of SCS.

Additionally, the lead author completed approximately 20 h of ethnographic observation. This occurred within the SCS spaces as well as in the surrounding neighbourhoods of each site. Observation and informal conversations were completed to gain a broader understanding of the social, structural, and physical contexts that may impact the implementation and uptake of SCS. The lead author also reviewed relevant policy documents, including SCS and police operations manuals. Field notes were taken to contextualize the research and to enhance the interview data [45].

The lead author reviewed the interview transcripts and met with the senior author to develop a coding framework using both a priori themes (e.g. fears of arrest, public drug use) and emerging themes (e.g. police discretion, police presence, lack of police presence) from the dataset [46]. NVivo 12—a qualitative data management software—was used to code the data into themes and subthemes for analysis [47]. Preliminary findings were presented to SCS management and staff as well as a community advisory group to solicit feedback and strengthen the validity of the findings.

## Results

The study sample included 24 participants between the ages of 20 and 64, with an average (mean) age of 37. Seventeen participants identified as men, with the remaining seven identifying as women. In terms of ethnicity, 13 participants were white and 11 were racialized and/or Indigenous. Fifteen participants had experienced at least one overdose in the last year. Participants had varied perspectives on, and experiences with, policing as they related to accessing SCS. The following themes related to policing of the SCS are discussed herein: (1) significant police presence, (2) fears of arrest, (3) lack of police presence, and (4) immunity from arrests. Additionally, a local police document on SCS provides context to these findings.

### Internal police report on SCS

Relevant policy documents pertaining to policing were reviewed to understand the policy context in which SCS operate. Included in this review was an official Toronto Police Service internal report on SCS and police activity [48]. The policy report discusses the Good Samaritan Drug Overdose Act that provides PWID with immunity from charges related to drug possession, conditions, and court orders thereby making it more likely that PWID will call emergency services (i.e. 9-1-1) in the event of an overdose. Additionally, this document provides an overview of federal law (e.g. the Control Drugs and Substances Act) that prohibits the possession of illicit drugs and how SCS have a federal exemption to protect staff and clients from the application of this legal subsection. Police are therefore unable to make arrests while individuals are inside SCS. While this legal exemption is not applicable to areas outside of the SCS, the document does state that policing in the vicinity could have negative impacts on access to SCS as well as hinder public health harm reduction strategies. However, the document repeatedly suggests that police officers can exercise discretion if they intend to investigate a client when they are travelling to and from an SCS. For example:

*While the position of the Toronto Police Service is that discretion should be considered so as to avoid a heightened police-presence, which could otherwise discourage potential service users of the sites from attending, it is still our duty to ensure the safe, free enjoyment of public spaces to all community members and stakeholders. This includes being accountable to all residents, visitors, businesses and community groups* [48].

Aside from exercising discretion when investigating SCS clients, the policy report emphasizes that the Toronto Police Service does not support or allow for a non-enforcement boundary in the vicinity of SCS and states that police should still enforce drug trafficking, which “would be deemed as a function that could reduce harm to others” [48].

#### Site #1: Significant police presence in the neighbourhood

Participants from each study site had varied experience with police presence in relation to accessing SCS. Participants from site #1 discussed a concentrated police presence in the neighbourhood and how they have had negative experiences with police related to their drug use. For example:

*I was a little mind blown by the amount of police presence but not so much in front of the building here. Like I mean like more on the corner and in the area today. Police are very familiar with who I am. Every division in Toronto knows me. They know I’m most likely to have drugs. Like my entire record is the distribution of narcotics, right?* (Participant 4, site #1)

Other participants described an inability to use drugs in the same neighbourhood without being targeted by police and discussed the need for safe spaces to use drugs—SCS or otherwise:

*Lots of police in this area. I think they should leave addicts alone. We need a place to be addicts. If you see us in the alley, don’t bother us, we’re trying to get out of the way. So, why do you come in the alley and jack me up?* (Participant 12, site #1)

Based on ethnographic observations in the neighbourhood of site #1, police encounters were frequent (e.g. witnessing patrols and arrests). While police were not observed outside of the SCS, they were seen in the immediate vicinity almost every time I left the SCS.

Aside from describing a police presence in the surrounding neighbourhood of site #1, some participants described seeing police parked in front of the SCS: “There are times when police are here…like just a week and a half ago, two police officers in a car come up, and they were sitting there” (participant 9, site #1). The presence of police in close proximity to the SCS, according to multiple participants, would impact their use of the SCS. For example, when asked about police presence, one participant said:

*Oh, I wouldn’t even come in. I would walk right past. Like see, I’m not in trouble for anything, but like it’s just the thing like they’ll just get ready to just stop you and talk to you for no reason. And nobody wants that.* (Participant 6, site #1)

When participants were asked about reasons why there are many people who use drugs in the surrounding neighbourhood, but less clients accessing SCS, the police were described as a notable barrier. For example:

*It’s the cops. A hundred percent it’s the cops. You’ll see them 20 times a day…The cops down here are known to be pricks and assholes, right? And they’re right up on certain people…There’s a couple of times I came and I wouldn’t even come in to get a kit. And I’m like, I see the police outside and I said, ‘no, no, no’ and I’d just stay away.* (Participant 11, site #1)

While participants had varying interactions with the police, all participants from site #1 described the police as having a negative impact on PWID within the neighbourhood. This is not surprising, given that during ethnographic observation, there was a noticeable presence of street activity (e.g. drug dealing, public injecting, signs of mental health distress) that likely increased policing and surveillance in the area.

#### Site #1: Fears of arrest when accessing SCS

Aside from having negative experiences with the police, participants from site #1 also described fears of arrest before, during, or after accessing SCS. This was sometimes described in terms of an overall anxiety related to police interactions as well as in relation to using at the SCS specifically. For example: “A lot of people have a lot of psychosis, or think they’re getting recorded, [or fearing that] cops are just coming in any time they want to, to search you” (participant 6, site #1). Paranoia was also discussed in reference to leaving the SCS: “When I leave sometimes I’m paranoid though. Sometimes I’m worried I’m going to get arrested on the way out” (participant 11, site #1). While paranoia was sometimes described as drug-induced (i.e. a result of crystal methamphetamine use), participants identified these experiences as warranted given the fact that using SCS immediately identifies individuals as PWID and may result in arrests. For example: “Well if cops will see us come in here and walking out of here, they know what we’re doing. They might want to catch us dealers” (participant 10, site #1). Participants discussed a need to be able to use drugs in the SCS without fears of arrest. As described by one participant:

*They’re outside all the time. Like, I asked the staff, right? like, I said, “What’s to stop them from stopping us when we leave here?” Right? To see if we finished all our dope and charging us with possession, right? You know, it’s, like, just leaving here, is that a probable cause for them to stop us? Like I didn’t know, right? I said, “Is there any way we can…we can have like a safety zone, like get a letter saying, you know, we got an hour to make it home or whatever, so they can’t stop us because we just left here?” Like, and she’s, like, “I don’t know, that’s a very good question.” Right? And what if they see you pick up and they know you’re coming here? Right? What’s to stop them from stopping you outside the door and charging you, right?* (Participant 11, site #1)

Given the observation of significant police presence in the neighbourhood surrounding site #1, and the targeting of PWID, participants’ descriptions of their fears of arrest when using SCS seem warranted rather than merely being a consequence of drug-induced paranoia.

#### Site #2: Lack of police presence

The perspectives and experiences of participants from site #2 regarding policing and the SCS were strikingly different. While participants did not have positive perspectives on the criminalization of PWID or policing in general, their experiences and perspectives on police in relation to the use of SCS at site #2 were opposite to those expressed by participants from site #1. Participants noted neighbourhood contexts as shaping the lack of police activity. For example: “You know, [this neighbourhood] is not a hotbed for crime” (participant 13, site #2). Participants also compared the neighbourhood activity to other SCS sites in Toronto. For example: “I don’t see any bike cops period around here. Around [site #1], every five minutes they’re circling around. But here, no. Even in their car it’s very rare” (participant 23, site #2). Participants also highlighted stark differences between site #2 and a downtown SCS, as demonstrated by the following quote:

*I find that this area in particular has a decreased police presence opposed to the one downtown…It’s a totally different neighbourhood, like how open people are to drug use downtown as opposed to here. Down here it’s like you’re more likely to see families and children…It’s just there’s not a lot of drug users openly using outside.* (Participant 16, site #2)

During 10 h of ethnographic observations over 3 days in the neighbourhood surrounding site #2, police were never encountered, and compared to site #1, there lacked a street presence of PWID (e.g. public injection, drug dealing). These observations highlight the contrasting drug neighbourhood drug scenes where one is a very open drug scene and the other is closed or hidden.

#### Site #2: SCS and immunity from arrests

Participants from site #2 not only discussed a lack of police presence within the neighbourhood, but in contrast to participants from site #1, they discussed perceived immunity from police arrests while using the SCS, as demonstrated in the following excerpts: “There’s nothing the cops can do” (participant 24, site #2), and “The only advantage about using there is that you can’t get busted from the police. So, I guess you could call it safe” (participant 14, site #2). Participant accounts highlighted the legality of injecting drugs at a SCS without fears of getting arrested. For example:

*It’s the only place where you feel safe. It’s the only place where you are legally allowed to use drugs…You come in here and you remain anonymous, you can come in here, you can use your drugs, and you can go. I don’t think there’s any reason to be fearful about coming here.* (Participant 18, site #2)

However, some participants did identify safety concerns and the potential for drug-related police arrests outside of the SCS: “It’d be safe here, but you know, 50 yards, 100 yards away, it’s not going to be safe” (participant 19, site #2). Further, when asked about this SCS compared to other sites, one participant suggested that PWID do not want to get caught possessing drugs and that they are “traveling and worrying about the cops” (participant 15, site #2). These perspectives highlight the realities of the criminalization of PWID regardless of the police presence in the neighbourhood.

## Discussion

In summary, participants had varying perspectives on, and experiences with, policing as they relate to accessing SCS. Participants from site #1 discussed the frequency of police in the area and the subsequent targeting of PWID. Police were often seen in the immediate vicinity and sometimes even in front of the SCS. Police activity in the neighbourhood created anxiety and fears of arrest when accessing SCS. Participants from site #2 discussed a lack of police presence and how its surrounding neighbourhood was different from other neighbourhoods (e.g. downtown), one that was more family-oriented and had less crime and street presence than downtown. These implementation contexts were also shaped by differences in the makeup of the neighbourhood drug scenes whereby one was open (site #1) and the other was closed (site #2). Participants also described how they felt safe using SCS given the immunity from arrest, though some discussed concerns about encountering police while travelling to and from the SCS with drugs in their possession. In addition, local police policies described the rights and protections of individuals within SCS, while also emphasizing the exercise of discretion afforded to officers regarding investigations and arrests of PWID outside of SCS.

Experiences of negative encounters with police by PWID have been well documented in international studies. The experiences of participants from site #1 are similar to past research that found that neighbourhood police presence and surveillance created fears and anxiety around potential arrests [29–31, 49]. Our findings are also consistent with studies on SCS access that have demonstrated police operating in the surrounding area as a barrier to uptake [50, 51] and overall fears of arrests via police warrants or media surveillance (e.g. cameras) [52]. While study participants from site #2 did not describe concerns for arrest while utilizing SCS, they did discuss how using these services provided them a space that was immune from arrest, which is similar to other studies that have found police avoidance to be a motivational factor for utilizing SCS [53–55].

While the SCS at each study site followed the same federal government operational guidelines and were similarly modelled and staffed, the intervention accessibility and experiences of clients were ultimately shaped by the distinct external implementation contexts of each SCS. Research on the implementation of a variety of HIV health interventions (e.g. testing, treatment) has considered the ways in which contextual factors can impact service delivery [56–58]. Hamilton et al. suggest that implementation research has focused more on internal rather than external contexts, and so despite internal organizational readiness for HIV interventions, external contexts can still negatively affect program implementation [58]. Others have highlighted the ever-changing information landscapes and the need for programs to be adaptable to these and how they are affected by social and structural forces [57]. Understanding how context either supports or limits implementation is thus critical to intervention success [56].

Drawing on our findings from each site, then, we can start to understand the effects of various contextual domains on the implementation of SCS [44]. Participants’ accounts, ethnographic observation, and reviews of policy documents highlight issues stemming from the geographical and legal contextual domains and the ways in which they interplay and impact SCS uptake. The geographical contexts are important to consider. Site #1 is in a neighbourhood of Toronto that is close to a variety of health and social services, including mental health services, drop-in spaces, and community centres, that generally serve PWID as well as other street-entrenched individuals. Consequently, these increase not only the street presence of individuals experiencing a variety of health-related challenges, but also the presence of police, especially given the open drug scene in this area. In the neighbourhood of site #2, however, there are less health and social services, no visible signs of street drug activity (e.g. drug dealing, public injection), and police presence was almost nonexistent. These contextual domains thus affect PWID differently in terms of their everyday interactions, including their access to SCS. Lack of police presence in the neighbourhood of site #2 was a facilitator for service access, while police presence in the neighbourhood of site #1 was seen as a barrier to service uptake. Considering these geographical and legal domains alongside public health interventions such as SCS thus provides a more robust understanding of the roles of these particular external contextual factors may have in shaping the outcomes and challenges of health interventions.

Community consultations prior to the implementation of SCS may not satisfy all stakeholders, and criticism and challenges from various groups are expected [19]. Implementation of SCS thus needs to account for other external contextual factors and include the development of strategies to ensure ongoing engagement with stakeholders from multiple contextual domains, including police. A recent study on SCS-police relationships highlights ways to develop cooperative relationships, including ongoing dialogue, dedicated police liaisons, and boundary agreements [38, 59]. SCS community consultations in Toronto identified diverging perspectives on the role of police, with some suggesting that a police presence may deter people from using SCS, and others wanting an increased police presence for neighbourhood safety [60].

While identifying the benefits of SCS for PWID, the Toronto Police Service policy report document states that officers can exercise discretion when investigating a potential SCS client, including when PWID are travelling to and from SCS [48]. However, the extent to which the recommendations from this report were implemented and practiced by police officers is not known. Still, it appears evident from ethnographic observation and participants’ accounts of policing in each neighbourhood that this exercise of discretion is not applied to all neighbourhoods and SCS equally, given the fact that police presence in the vicinity of site #2 was nonexistent. Studies in other jurisdictions have highlighted how despite the fact that there may be some laws protecting PWID, the use of police discretion is often inconsistent and some PWID continue to be targeted [51, 61]. This demonstrates not only the police profiling of PWID, but also the gaps between translating policy and laws into policing practice [61]. Unfortunately, the Toronto Police Service policy report was created internally without any consultation with SCS management or staff. While educating police and developing cooperative SCS-police relationships is a start [38, 41, 61], PWID will continue to face police-created barriers to access given the fact that they are criminalized [24]. Future SCS implementation should not only consider developing positive SCS-police relationships, but also more importantly, PWID-police relationships and how criminalization and other socio-structural implementation contexts constrain SCS access. Additionally, the creation of a non-enforcement boundary outside of SCS warrants further investigation.

There are some limitations to this study. First, while we made an effort to reach a diversity of participants, their perspectives and experiences may not be reflective of all SCS clients from each site. Second, we did not interview police regarding their practices surrounding the use of discretion and policing in close proximity to SCS. Third, we did not examine other external implementation contexts, so there may be other domains that interplay with police that are either barriers or facilitators in shaping access to SCS. Therefore, future research should investigate these other contextual domains (e.g. epidemiological, ethical, political, socio-cultural, and socio-economic) and their effects on the implementation of SCS.

In conclusion, a comparison of the participants’ perspectives of, and experiences with, police from two different sites impacted SCS access. It is evident that external barriers and facilitators, including neighbourhood context, laws, and policies, as well as SCS/police relationships play important roles in shaping access to SCS. Until larger structural implementation barriers are addressed (e.g. criminalization), future SCS programming should consider the impact of policing on the SCS implementation context to improve client experience with, and access to, these innovative harm reduction programs.
